# Machine learning-based prediction of postoperative venous thromboembolism in orthopedic surgery using the MIMIC-IV database and external validation

**DOI:** 10.3389/fsurg.2026.1884949

**Published:** 2026-09-17

**Authors:** Genfa Wu, Gengjiao Cai, Gang Wu, Minhua Zhu, Jinxing Guo

**Affiliations:** Department of Orthopedic Surgery, Yingtan 184 Hospital, China RongTong Medical Healthcare Group Co. Ltd., Yingtan, Jiangxi Province, China

**Keywords:** D-dimer, machine learning, MIMIC-IV database, orthopedic surgery, venous thromboembolism

## Abstract

**Objective:**

A risk prediction model for venous thromboembolism (VTE) after orthopedic surgery was constructed and validated using machine learning algorithms, providing a reference for the early identification of high-risk individuals in clinical settings.

**Method:**

Patients who underwent orthopedic surgery were retrospectively collected from the Medical Information Mart for Intensive Care (MIMIC-IV) database as the modeling group. The patients were randomly divided into training and internal test sets in a 7:3 ratio. A total of 156 patients who underwent orthopedic surgery in our hospital from January 2022 to July 2025 were selected for the external validation set. Predictive variables were selected using the least absolute shrinkage and selection operator (LASSO) regression. Six machine learning models were constructed: logistic regression (LR), decision tree (DT), random forest (RF), extreme gradient boosting (XGB), light gradient boosting machine (LGBM), and naive Bayes (NBM). The model performance was evaluated by using the area under the receiver operating characteristic curve (AUC), accuracy, sensitivity, specificity, F1 score, and G-mean. The interpretability of the optimal model was analyzed using the calibration curves, decision curve analysis (DCA), and SHAP methods.

**Results:**

The MIMIC-IV database included 461 patients (VTE incidence rate, 15.8%). LASSO regression identified two predictive variables: fibrinogen and D-dimer levels. The AUC of the LGBM model in the internal test set was 0.893 (95% CI: 0.820–0.966), and in the external validation, the AUC of the LGBM model was 0.788. The predictive performance of this model was superior to that of the other five models tested. The calibration curve indicated that the model was well calibrated, and the decision curve suggested a clinical net benefit within the 0.1–0.6 threshold range. SHAP analysis showed that elevated fibrinogen and D-dimer levels were positively correlated with the risk of VTE.

**Conclusion:**

The LGBM model constructed based on the MIMIC-IV database has excellent predictive efficacy for VTE after orthopedic surgery and certain external generalization abilities. Fibrinogen and D-dimer levels are key predictive factors. This model can serve as a preliminary reference tool for risk stratification of VTE after orthopedic surgery.

## Introduction

Venous thromboembolism (VTE) encompasses two major categories: deep venous thrombosis (DVT) and pulmonary embolism (PE) (1). Studies have shown that all patients undergoing major orthopedic surgeries are at a high risk of developing VTE (2). Among them, fatal PE is an important cause of unexpected death during the perioperative period and in the hospital. A study reported that the incidence of thromboembolic complications after major orthopedic surgeries is as high as 36%–60% (3), which greatly endangers the medical safety of orthopedic surgeries. Identifying and evaluating post-surgical VTE in orthopedics in an early, comprehensive, and effective manner has become an urgent issue. The disease risk prediction model, as a quantifiable risk assessment tool, is of great significance for the early identification of disease-influencing factors and high-risk groups. As the application of machine learning in the medical field continues to deepen, an increasing number of researchers are using various machine learning modeling methods to conduct research on patients undergoing various orthopedic surgeries. They integrate and analyze clinical data using big data algorithms, build models to predict potential risks, reduce the incidence of VTE, and improve patient prognosis (4). The Medical Information Mart for Intensive Care IV (MIMIC-IV) database of the United States is an electronic medical record database under PhysioNet (5). It covers the entire process data of patients with various diseases during their treatment, and the case resources are abundant. This study aimed to develop and validate a machine learning prediction model for predicting the risk of VTE after orthopedic surgery based on the MIMIC-IV database and evaluate the generalization ability of the model using an external dataset, with the expectation of providing a new reference tool for individualized risk assessment of VTE after orthopedic surgery.

## Materials and methods

### General information

The data for this study were derived from the clinical records of inpatients who underwent orthopedic surgery in the MIMIC-IV database (6). Specifically, patients who underwent orthopedic surgeries (including hip/knee joint replacement surgeries, fracture internal fixation surgeries, and spinal fusion surgeries) were selected by screening based on ICD-9 surgery codes (810, 811, 814, 815, 816, and 790) and ICD-10 codes (0RBG, 0RBH, 0SBB, 0SBC, 0SBD, and 0SBE). To ensure data accuracy, all patients who underwent orthopedic surgery during their hospital stay were included. The authors of this study completed relevant courses (including nine modules such as ethics, biosafety, and data management) on the official website of the database and passed the assessment. They obtained permission to access the database from the relevant institution (certificate number: 76515080). As MIMIC-IV is an anonymous database, patient informed consent was waived. Simultaneously, patients who underwent orthopedic surgeries at our hospital from January 2022 to July 2025 were selected as the external validation set. All patients provided informed consent. This study was approved by the ethics committee of our hospital.

### Inclusion and exclusion criteria

The inclusion and exclusion criteria for the external validation set were consistent with those of the modeling group. The definitions of all predictor variables, the time window for laboratory index testing (within 24 h before surgery), and the criteria for determining VTE outcomes were the same as those of the MIMIC-IV modeling group. The inclusion criteria were as follows: (1) patients underwent orthopedic surgery; (2) no coagulation disorders; and (3) complete clinical visit records and follow-up data of the patients were available. The exclusion criteria were as follows: (1) patients with VTE before surgery; (2) patients who received anticoagulant treatment before surgery; and (3) patients with a missing rate of key variables exceeding 20%.

### Data collection

This study extracted clinical data from the MIMIC public database and the hospital's electronic system, including: (1) basic information: age, sex, smoking history, drinking history, history of hypertension, diabetes, kidney disease, and cerebrovascular disease; (2) complications: VTE, including DVT and PE, were coded using ICD. The time window for VTE occurrence was defined as the period from after the surgery until before discharge, during which new diagnoses of DVT or PE were made. The diagnosis of DVT and PE was based on ICD codes recorded in the discharge diagnosis, and the diagnosis time must be later than the surgery date. Deep vein thrombosis: 45,111, 45,119, 4,512, 4,519, 452, 453 (ICD 9); I801, I820, (ICD 10), PE: 41,511, 41,519 (ICD 9); I260, I2609 (ICD 10); (3) physiological indicators: heart rate (HR), respiratory rate, systolic blood pressure (SBP), diastolic blood pressure (DBP), and mean blood pressure (MBP); And (4) biochemical indicators: white blood cell (WBC) count, platelets, serum creatinine (SCr), blood urea nitrogen (BUN), fibrinogen, D-dimer, and haemoglobin (Hb) within 24 h before the operation. All laboratory indicators were collected within 24 h before the operation. For patients with D-dimer and fibrinogen levels above the normal upper limit, the researchers checked the electronic medical record system to determine whether DVT or PE had been diagnosed before the operation. If DVT or PE had been diagnosed before the operation, they were excluded according to the exclusion criteria.

### Feature selection

All candidate predictive factors were clinical and laboratory indicators collected within 24 h before the operation, ensuring their applicability for preoperative risk assessment. During the data preprocessing stage, variables with a missing rate greater than 20% were eliminated, and for continuous variables with a missing rate of 20% or less, multiple imputation methods were used to fill in missing values. First, the MIMIC-IV modeling group was randomly divided into a training set and a test set in a 7:3 ratio. Then, a multiple imputation model was fitted solely based on the data from the training set, and this imputation model was applied separately to the training set and the internal test set. All data-based preprocessing steps (including LASSO feature selection, model training, and hyperparameter tuning) are completed within the training set. The internal test set and external validation set are only used for the final performance evaluation. During the construction of the prediction model, LASSO regression was employed for feature selection. The LASSO feature selection is strictly conducted within the training set. It fits and selects the model based on the data of the training set, and the selection process is completely unaffected by the distribution of the test set. LASSO effectively compresses the coefficients of insignificant variables to zero by introducing L1 regularization, thereby selecting the variables that have the greatest impact on the prediction results (7). In this study, all potential predictors (including demographic information and laboratory indicators) were incorporated into the LASSO model, and the optimal *λ* value was selected based on 10-fold cross-validation to ensure the robustness and predictive ability of the model.

### Model construction

Based on the selected feature variables, six machine learning algorithms were used to build prediction models including decision tree (DT), random forest (RF), extreme gradient boosting (XGB), light gradient boosting machine (LGBM), naïve Bayes model (NBM), and logistic regression (LR). XGB employs a random search combined with 5-fold cross-validation and determines the optimal number of iterations through an early stopping mechanism. LightGBM determines the optimal number of iterations through 5-fold cross-validation, whereas other parameters use default values. LR, DT, RF, and NBM used the default parameters of the R package. The MIMIC-IV dataset was randomly divided into a training set and an internal test set in a 7:3 ratio, respectively. The clinical data of hospitalized patients were used as an external validation set. During the training process, 5-fold cross-validation was performed to reduce the risk of model overfitting. The model performance was evaluated using an internal test set to ensure the generalization ability of the model. During the cross-validation process of hyperparameter tuning, the interpolation parameter fitting and LASSO feature selection are strictly nested within each fold.

### Model evaluation

The predictive performance of the model was evaluated by comparing accuracy, sensitivity, specificity, precision, F1 score (8), G-mean, and area under the curve (AUC). Decision curve analysis (DCA) and calibration curves were used to compare the clinical practical value and calibration degree of the model. The Shapley additive explanations (SHAP) model was adopted to explain the model and determine the contribution of each features to the risk of VTE, thereby further enhancing the transparency and clinical interpretability of the model.

### Statistical methods

Data analysis was conducted using SPSS 23.0 and RStudio 4.5. To ensure the integrity and accuracy of the research data, variables with a missing value ratio exceeding 20% were deleted, and for variables with a missing value ratio of less than 20%, multiple imputation methods were used for imputation. The normality of the measurement data was tested using the K-S test. Measurement data that followed a normal distribution are presented as mean ± standard deviation $\left(\bar{x}\pm s\right)$, and the differences between groups were compared using the t-test. Measurement data that do not follow a normal distribution are expressed as M (P25, P75), and comparisons between groups were conducted using the rank sum test. The count data are presented as the number of cases and percentage [*n* (%)], and the comparison between groups was performed using the *χ*^2^ test. Dfferences were considered statistically significant at *p* < 0.05.

## Result

### Baseline characteristics of the study population

After screening, 461 patients were included in the MIMIC-IV database as the modeling group, among whom 73 (15.84%) developed VTE after surgery. The external validation set included 156 patients, and the VTE incidence rate was 19.87% (31/156). The modeling group was randomly divided into training and internal test setsin a 7:3 ratio. There were no statistically significant differences in the baseline characteristics between the two groups (*p* > 0.05). The majority of baseline indicators in the modeling group and the external validation set showed statistically significant differences (*p* < 0.05); this difference originated from the inpatient population of multiple general hospitals in MIMIC-IV, whereas the external validation set was stratified by patients in general wards and orthopedic departments. This objectively led to the fundamental stratification of baseline thrombus levels and coagulation indicators, which would weaken the generalization effect of the model across different populations and could only be used as an independent external sample for preliminary efficacy testing. Details are shown in Tables 1, 2.

**Table 1:** Baseline data of patients in the training set and the internal test set.

Variable	Internal test set (*n* = 139)	Training set (*n* = 322)	*Z*/*χ*^2^	*p*
Gender [*n* (%)]			0.017	0.895
Female	70 (50.36)	160 (49.69)		
Male	69 (49.64)	162 (50.31)		
Age (years)	64.01 (47.42, 83.43)	66.63 (52.40, 83.33)	0.618	0.537
Smoking [*n* (%)]	12 (8.63)	33 (10.25)	0.288	0.592
Drinking alcohol [*n* (%)]	3 (2.16)	3 (0.93)	0.383	0.536
Hypertension [*n* (%)]	59 (42.45)	127 (39.44)	0.364	0.546
Diabetes [*n* (%)]	26 (18.71)	50 (15.53)	0.712	0.399
Renal disease [*n* (%)]	15 (10.79)	19 (5.90)	3.399	0.065
Cerebrovascular disease [*n* (%)]	6 (4.32)	16 (4.97)	0.091	0.763
HR (bpm)	90.00 (79.00, 104.00)	94.00 (80.75, 106.00)	1.037	0.300
Resp rate (bpm)	18.00 (16.00, 22.00)	18.00 (16.00, 22.00)	0.767	0.443
SBP (mmHg)	127.00 (112.00, 145.00)	123.00 (106.00, 142.00)	1.746	0.081
DBP (mmHg)	68.00 (55.00, 82.00)	66.50 (54.00, 78.00)	1.084	0.278
MBP (mmHg)	85.00 (71.00, 97.00)	80.00 (67.00, 93.00)	1.950	0.051
WBC (×10^9^/L)	11.20 (8.00, 14.80)	10.95 (8.90, 14.20)	0.117	0.907
Platelets (×10^9^/L)	211.00 (162.00, 272.00)	203.00 (162.00, 250.25)	1.393	0.164
SCr (mg/dL)	0.80 (0.70, 1.20)	0.90 (0.70, 1.10)	0.007	0.994
BUN(mg/dL)	16.00 (11.00, 23.00)	17.00 (12.00, 23.00)	1.090	0.276
Fibrinogen (g/L)	3.23 (2.66, 3.74)	3.24 (2.63, 3.86)	0.188	0.851
D dimer (µg/mL)	1.55 (1.30, 1.88)	1.64 (1.33, 1.92)	1.179	0.238
Hemoglobin (g/L)	11.20 (9.70, 12.50)	11(9.70, 12.10)	0.939	0.348

**Table 2:** Baseline data of patients in the modeling group and the external validation set.

Variable	Modeling group (*n* = 461)	External validation set (*n* = 156)	*Z*/*χ*^2^	*p*
Gender [*n* (%)]			0.281	0.596
Female	230 (49.89)	74 (47.44)		
Male	231 (50.11)	82 (52.56)		
Age (years)	65.25 (51.14, 83.33)	57.50 (51.00, 64.00)	5.126	<0.001
Smoking [*n* (%)]	45 (9.76)	28 (17.95)	7.490	0.006
Drinking alcohol [*n* (%)]	6 (1.30)	15 (9.62)	24.505	<0.001
Hypertension [*n* (%)]	186 (40.35)	59 (37.82)	0.311	0.577
Diabetes [*n* (%)]	76 (16.49)	48 (30.77)	14.808	<0.001
Renal disease [*n* (%)]	34 (7.38)	31 (19.87)	19.312	<0.001
Cerebrovascular disease [*n* (%)]	22 (4.77)	23 (14.74)	17.140	<0.001
HR (bpm)	93.00 (80.00, 106.00)	95.00 (85.00, 107.00)	1.813	0.070
Resp rate (bpm)	18.00 (16.00, 22.00)	20.00 (17.00, 23.00)	3.582	<0.001
SBP (mmHg)	125.00 (108.00, 143.00)	126.00 (109.25, 138.00)	0.165	0.869
DBP (mmHg)	67.00 (55.00, 79.00)	70.00 (60.25, 81.00)	1.877	0.060
MBP (mmHg)	81.00 (68.00, 94.00)	80.50 (70.25, 92.00)	0.381	0.703
WBC (×10^9^/L)	11.00 (8.60, 14.30)	12.70 (10.60, 15.17)	3.474	0.001
Platelets (×10^9^/L)	205.00 (162.00, 259.00)	214.00 (194.00, 237.00)	1.662	0.097
SCr (mg/dL)	0.80 (0.70, 1.10)	1.48 (1.26, 1.81)	13.123	<0.001
BUN (mg/dL)	17.00 (12.00, 23.00)	21.00 (17.00, 24.00)	4.630	<0.001
Fibrinogen (g/L)	3.24 (2.65, 3.79)	3.50 (2.70, 4.20)	2.345	0.019
D dimer (µg/mL)	1.60 (1.32, 1.90)	1.69 (1.39, 2.03)	1.821	0.069
Hemoglobin (g/L)	11.10 (9.70, 12.20)	10.60(9.30, 11.57)	3.107	0.002

### Variable selection

The 20 variables included in the study were subjected to Lasso regression screening. The *λ* value was calculated using 10-fold cross-validation with Lasso-CV. LASSO feature selection was conducted only in the training set, and two non-zero coefficient variables were selected: fibrinogen and D-dimer. Details are shown in Figure 1.

**Figure 1:**
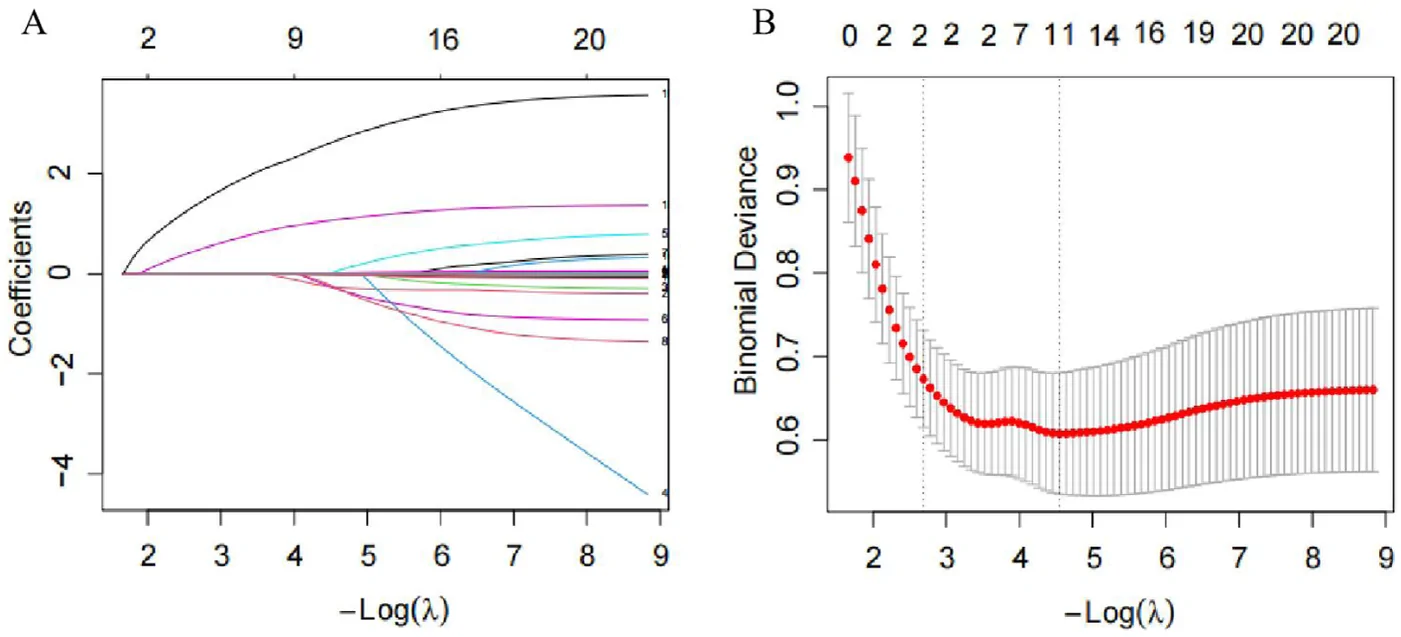
Results of variable selection and parameter optimization in LASSO regression. (**A**) Changes in LASSO regression coefficients, the *x*-axis represents Log Lambda, the *y*-axis represents the regression coefficients. Different colored lines correspond to the coefficient variations of each analysis variable at different Log Lambda values; (**B**) Curves of binomial deviation in LASSO regression with respect to Log Lambda changes, used to assist in selecting the optimal regularization parameter.

### Model construction and validation

The occurrence of VTE in postoperative patients was considered the dependent variable (0 = no occurrence, 1 = occurrence). The two influencing factors selected by the LASSO regression were used as independent variables for the model construction. Variable assignment: Fibrinogen and D-dimer were both input as their original values. The predictive performance of six machine learning algorithms, including LR, DT, RF, XGB, LGBM, and NBM, was compared. The results show that the AUC of the LGBM model in the internal test set was 0.893 (95% CI: 0.820–0.966), which was higher than that of LR (0.865), DT (0.803), RF (0.890), XGB (0.888), and NBM (0.885). Although NBM outperformed LGBM in terms of accuracy (89.9%), precision (55.0%), and F1 score (0.611), its sensitivity was only 68.8%, the lowest among the six models, suggesting that NBM might have sacrificed the detection of VTE-positive patients for a higher negative predictive accuracy. Considering the AUC value, sensitivity, and external validation performance, this study ultimately selected LGBM as the optimal model. The ROC curves for each machine learning algorithm are shown in Figure 2. The accuracy, sensitivity, and F1 values of each model are detailed in Table 3. The external dataset was verified using the LGBM model. The results showed that the AUC of the LGBM model for external validation was 0.788, indicating that the LGBM model constructed in this study had a strong ability for external generalization.

**Figure 2:**
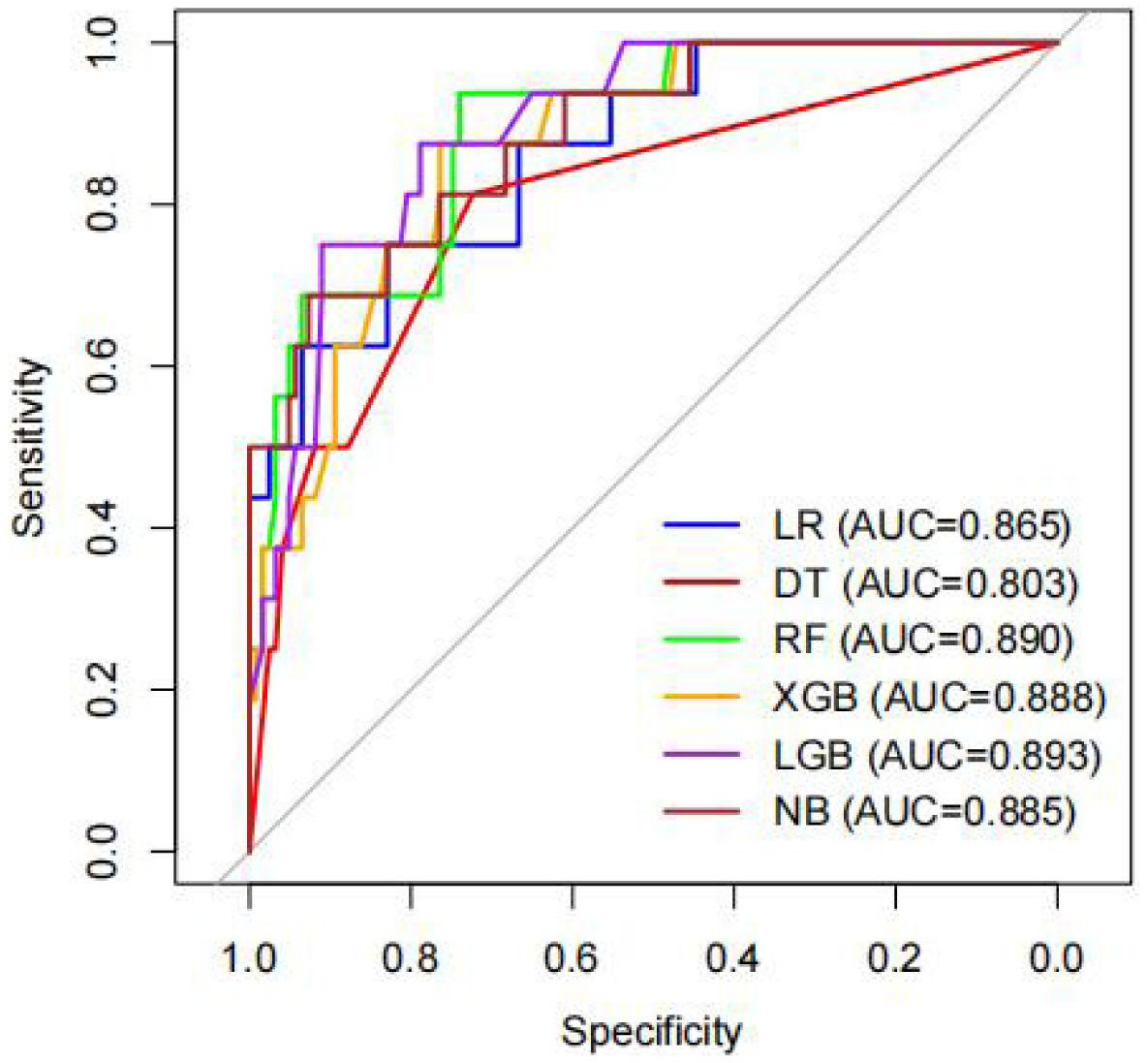
ROC curve diagrams of six machine learning models. LR, logistic regression; DT, decision tree; RF, random forest; LGBM, light gradient boosting machine; NBM, Naïve Bayes model; XGB, extreme gradient boosting; AUC, area under the curve.

**Table 3:** Comparison of prediction performance indicators of six machine learning algorithms.

Model	AUC	95%CI	Accuracy (%)	Sensitivity (%)	Specificity (%)	Precision (%)	F1 score	G-mean
LR	0.865	0.771–0.959	0.820	0.750	0.829	0.340	0.490	0.789
DT	0.803	0.684–0.922	0.734	0.812	0.724	0.277	0.413	0.767
RF	0.890	0.811–0.969	0.763	0.938	0.740	0.319	0.476	0.833
XGB	0.888	0.809–0.966	0.799	0.875	0.789	0.350	0.500	0.831
LGBM	0.893	0.820–0.966	0.799	0.875	0.764	0.326	0.475	0.818
NBM	0.885	0.798–0.972	0.899	0.688	0.927	0.550	0.611	0.798
External verification	0.788	0.678–0.898	0.692	0.710	0.688	0.361	0.478	0.699

LR, logistic regression; DT, decision tree; RF, random forest; LGBM, light gradient boosting machine; NBM, Naïve Bayes model; XGB, extreme gradient boosting; AUC, area under the curve.

### Optimal model evaluation

The accuracy of the LGBM model's prediction probabilities was evaluated using a calibration curve, and a decision curve assessed the clinical net benefits of the model at different decision thresholds. The calibration curve closely followed the diagonal line in most areas, indicating that the model's predicted probabilities were consistent with the actual event occurrence rates, demonstrating good calibration performance. The Brier score was 0.094, suggesting that the model's prediction error on the internal dataset was small and its calibration performance was good. The DCA curve shows that the LGBM model has a relatively high clinical net benefit within the decision threshold range of 0.1–0.6 (Figure 3).

**Figure 3:**
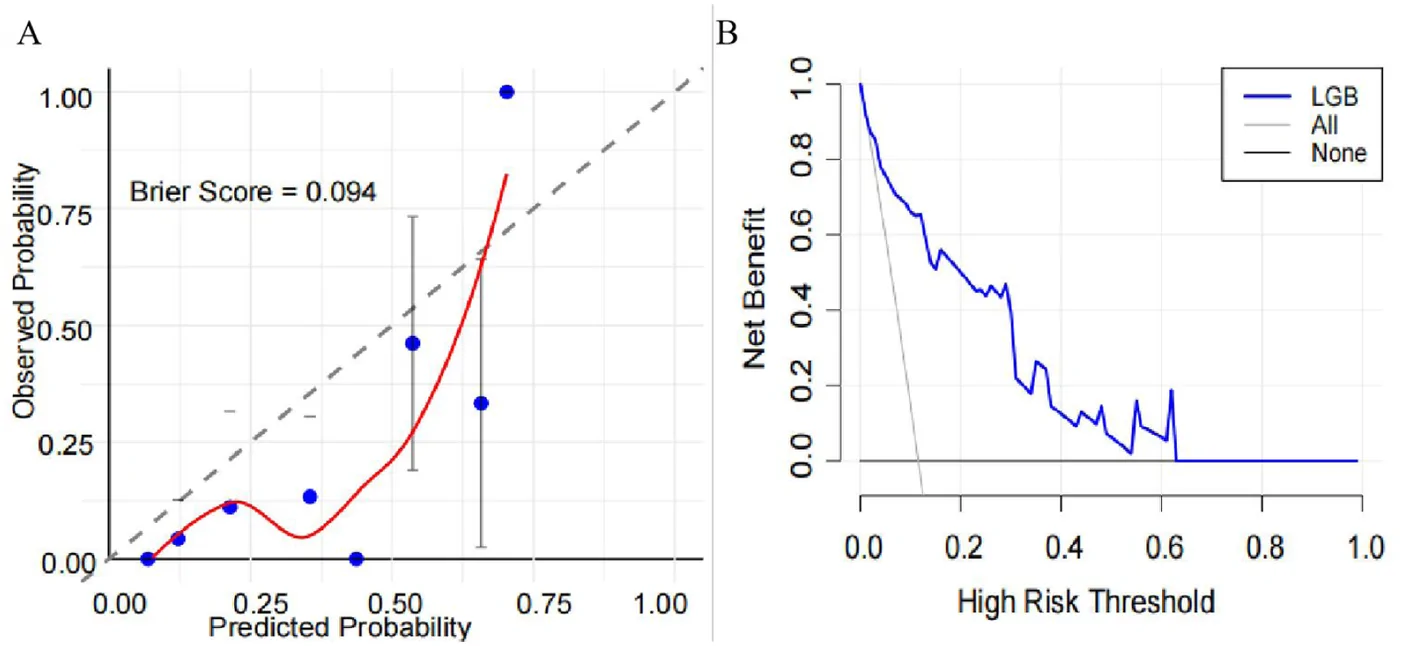
Calibration curve (**A**) and DCA curve (**B**) of the LGBM model in internal validation. (**A**) Calibration curve of LGBM model in internal validation; (**B**) Clinical DCA of LGBM model in internal validation.

### External verification

The optimal LGBM model was directly applied to an external validation set. The results (last row of Table 3) show that the external validation AUC of the model was 0.788 (95% CI: 0.678–0.898), with accuracy of 69.2%, sensitivity of 71.0%, specificity of 68.8%, and F1 score of 0.478. The positive predictive value (accuracy rate) was 36.1%, indicating that the model's performance in predicting the positive category was limited. This result suggests that although the model performed reasonably well in terms of overall discrimination ability (AUC), the relatively low positive predictive value in actual clinical applications may lead to a certain proportion of over-warning, and it should be used with caution in combination with clinical decision-making. The calibration of the model was evaluated using the Brier score (9). A lower score indicates better calibrations. The Brier score of the LGBM model in the external validation set was 0.136, suggesting good calibration. The DCA curve showed that the LGBM model had a high clinical net benefit within the decision threshold range of 0.1–0.6 (Figure 4).

**Figure 4:**
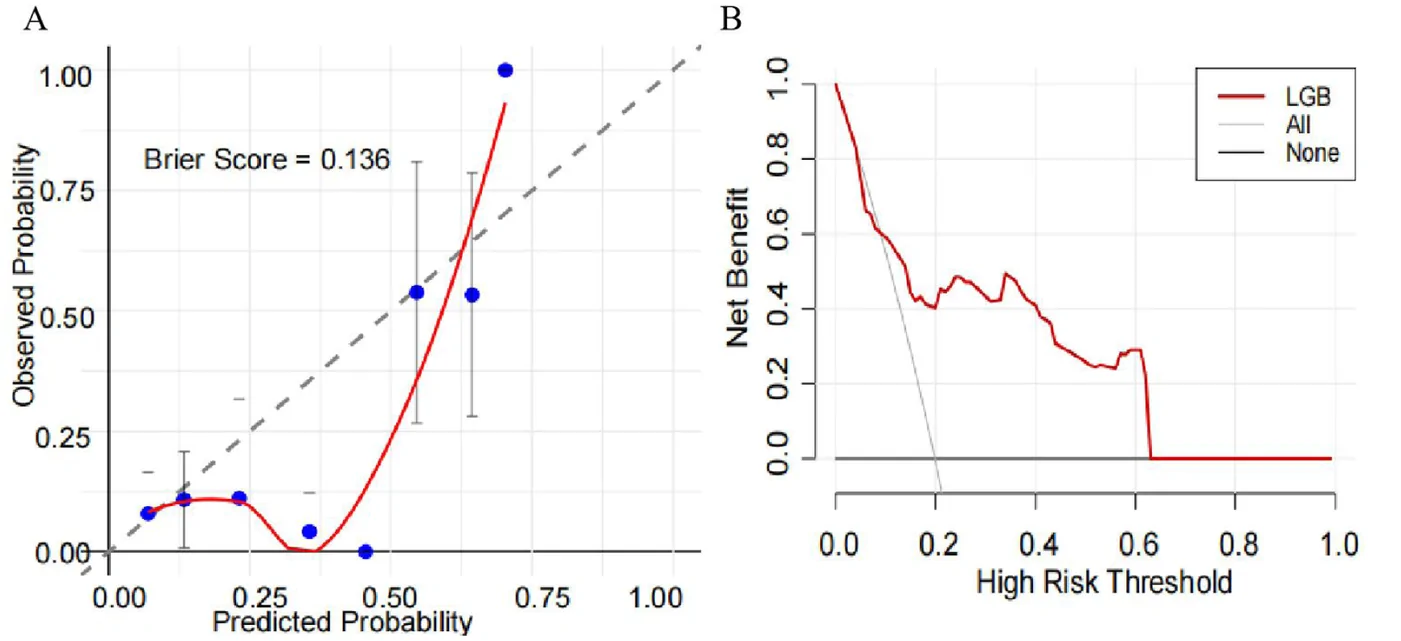
Calibration curve **(A)** and DCA curve **(B)** of the LGBM model in external validation.

### Optimal model explanation

The SHAP algorithm ranks and scores the contribution values of each variable to the outcome event, quantifying the importance of each variable in predicting the risk of VTE.A positive SHAP value indicates that this feature value points towards the direction of “VTE occurrence” (high risk), while a negative value points towards the direction of “VTE non-occurrence” (low risk). Since LASSO regression retained only fibrinogen and D-dimer as the two variables after screening, the SHAP analysis only included these two features. The SHAP scatter plot (Figure 5) illustrates the contributions of fibrinogen and D-dimer levels in the LGBM model to the prediction of postoperative VTE risk. The ranking of average absolute SHAP values indicated that fibrinogen had the greatest contribution (0.075), followed by D-dimer (0.065). The SHAP scatter plot indicates that elevated levels of fibrinogen (g/L) and D-dimer (µg/mL) (in the purple area) mainly pushed the SHAP value towards positive values, suggesting a positive correlation with the postoperative VTE risk.

**Figure 5:**
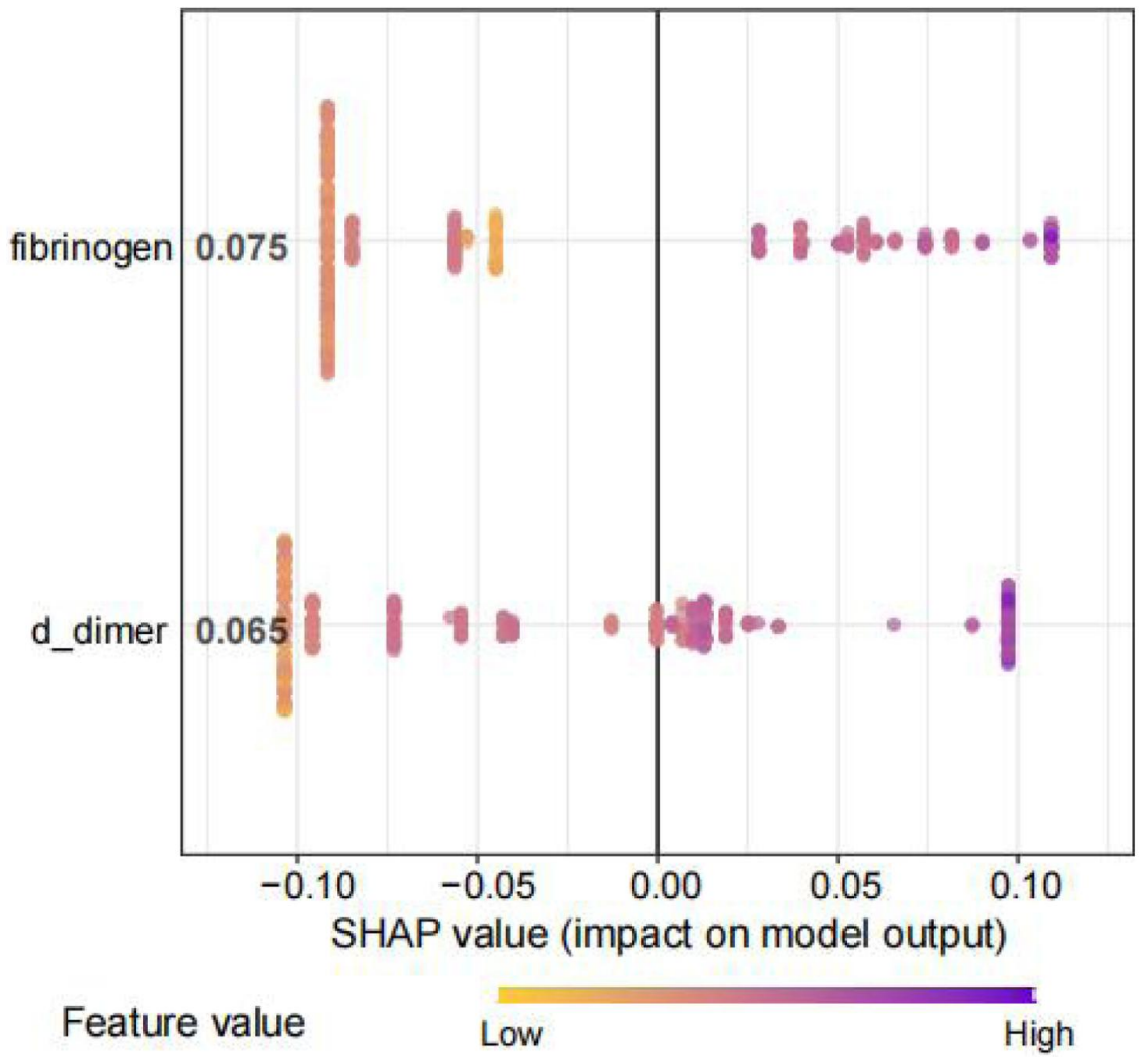
SHAP analysis.

## Discussion

The occurrence of VTE has multiple causes, mainly related to the patient's venous blood flow, venous wall, and blood conditions, including sluggish venous blood flow, damaged venous walls, and hypercoagulable blood states (10). Patients undergoing major orthopedic surgery are at a high risk of VTE. Postoperative VTE is a major cause of unexpected death during the perioperative period (11–13). Early identification of high-risk patients and implementation of individualized anticoagulation prevention are key to improving prognosis.

In recent years, researchers have developed various VTE assessment scales and clinical prediction models (14–16). From a technical perspective, previous studies mostly employed traditional logistic regression analysis for modeling, which has strong universality but is less targeted for predicting VTE in critically ill patients. Moreover, it is difficult to avoid limitations, such as limited inclusion of risk factors and small room for subsequent improvement (17). With the rapid development of the Internet and big data industry, machine learning, as a new data analysis tool, has entered the medical field and is widely applied in areas such as disease diagnosis and medical image recognition (18). It has unique advantages in handling large-sample data and complex tasks and has become a powerful tool for analyzing large amount of medical health data and improving clinical decision-making ability (19). This study constructed a prediction model for postoperative VTE in orthopedic patients based on machine learning methods and evaluated the model's performance using an external validation set. The research results show that the LGBM model constructed based on the preoperative levels of fibrinogen and D-dimer achieved an AUC of 0.893 in the internal test and 0.788 in the external validation. This preliminary verification demonstrates the potential application of this model in the risk stratification of VTE after orthopedic surgery.

This study was based on the MIMIC-IV database and systematically compared the efficacy of six machine learning algorithms in predicting the risk of VTE after orthopedic surgery. External validation was performed using independent hospital datasets. The results showed that the LGBM model performed the best in the internal test set (AUC = 0.893, 95% CI: 0.820–0.966) and still had good discrimination ability in the external validation (AUC = 0.788). LASSO regression selected fibrinogen and D-dimer as the two non-zero coefficient variables from the 20 candidate variables, suggesting that they are predictors of VTE after orthopedic surgery. The LASSO regression only selected two variables, fibrinogen and D-dimer, reducing the necessity of using complex nonlinear models. Compared with LR (AUC = 0.865), LGBM (AUC = 0.893) improved the AUC by only 0.028. Moreover, in the external validation, the difference between the two models further narrowed, suggesting that in cases where the number of predictor variables is limited, the gains of complex machine learning models over traditional logistic regression are limited. Simple models are often sufficient to provide good predictive performance, whereas complex models increase the risk of overfitting. Nevertheless, this study chose LGBM as the final model, mainly due to its slight advantage in AUC in the internal test set and the convenience of SHAP interpretability analysis. Future research should incorporate more dimensions of predictive variables to fully leverage the advantages of machine learning in large-scale and high-dimensional data.

Fibrinogen not only participates in thrombus formation as a substrate in the coagulation cascade reaction but also promotes platelet aggregation by binding to platelet surface receptors (GPⅡb/Ⅲa), increasing blood viscosity. At the same time, fibrinogen is an acute-phase reactant protein, and its elevation often accompanies an inflammatory state. Inflammation and thrombosis have a thrombo-inflammatory interaction, which further amplifies the risk of thrombosis (20). Several studies conducted on the Chinese population have confirmed this association. A cohort study (*n* = 126) of patients after hip joint replacement under general anesthesia showed that the serum fibrinogen level in the DVT group was significantly higher than that in the non-DVT group. Logistic regression analysis confirmed that serum fibrinogen (OR = 1.859, 95% CI: 1.176–2.939) was a risk factor for DVT after HA surgery (21). Another retrospective analysis of venous thromboembolism (VTE) during the perioperative period in patients with spinal fractures showed that the fibrinogen level in the VTE group was significantly higher than that in the non-VTE group, and plasma fibrinogen ≥3.75 g/L was positively correlated with VTE (22). D-dimer is a specific degradation product of crosslinked fibrin. An increase in its level directly indicates the presence of active thrombosis and secondary fibrinolysis (23). In a study by Dong et al. (24), the serum D-dimer level in the VTE group was significantly higher than that in the non-VTE group. Multivariate analysis revealed that D-dimer was also an independent risk factor for VTE in patients with traumatic lower limb fractures (OR = 1.140, 95% CI: 1.085–1.198). Luo et al. (25) conducted a prospective study on DVT after total knee arthroplasty and found that for patients with a preoperative Caprini score of ≥4 and a D-dimer level of ≥1.0 mg/L, the postoperative DVT incidence was as high as 24.81%, with an OR of 4.744.

In terms of model performance, the AUC of the LGBM model in the internal test set was 0.893, indicating that its predictive performance was superior to that of the other models. The external validation AUC of the LGBM model was 0.788, which was approximately 0.105 lower than that of the internal test set (0.893). The accuracy (69.2%) and F1 score (0.478) were also relatively low, suggesting that the generalization ability of the model across datasets is limited. This may be related to the significant differences in baseline features between the modeling and external validation groups This result is consistent with those of other studies on the prediction of VTE in orthopedics. In the study by Baki et al. (26) on the prediction of DVT after tibial fracture surgery, by combining 42 algorithm-feature selection combinations, it was found that various models such as Random Forest and LGBM achieved an AUC range of 0.97–0.99. A study on VTE risk prediction for orthopedic inpatients showed that the XGB model achieved the highest AUC of 0.931, significantly outperforming the traditional Caprini scoring model (27). SHAP analysis further confirmed that elevated levels of fibrinogen and D-dimer were positively correlated with the risk of VTE, and the contribution of fibrinogen was slightly greater than that of D-dimer. The calibration curve and decision curve analyses showed that the model had good calibration performance and clinical net benefit. These findings provided a new quantitative tool for the individualized risk assessment of VTE after orthopedic surgery.

However, this study still has certain limitations. First, although the MIMIC-IV database includes all hospitalized patients rather than only ICU patients, it is derived from a single medical center in the United States. The racial composition of patients, their treatment habits, and the baseline risk of VTE differ from those of the Chinese population. This may affect the applicability of the model in the Chinese orthopedic surgery population. Furthermore, the MIMIC-IV database contains retrospective electronic medical record data, and the completeness and accuracy of some key perioperative variables were affected by the quality of the original records. Second, VTE outcomes were identified based on ICD codes, which may lead to coding errors or omissions, and there is a lack of precise records of the occurrence time of postoperative VTE. Third, the retrospective design may have selection bias. Key perioperative variables (such as type of surgery, duration of surgery, and intraoperative blood loss) were not included, which weakened the persuasiveness of the model. Fourth, VTE, as an imbalanced outcome (with an incidence rate of 15.8% in the modeling group), suffers from a class imbalance issue, which may limit the model's ability to identify the positive category (external validation F1 = 0.478). Fifth, the sample size for external validation was relatively small (*n* = 156) and was derived from only one center. The applicability of the model in hospitals of different levels and populations across different regions requires further verification. Sixth, although this study excluded patients with preoperative diagnosed VTE through ICD coding and medical record verification, due to the inherent limitations of the retrospective database, we were unable to conduct routine Doppler ultrasound screening for asymptomatic patients with elevated D-dimer or fibrinogen levels before the operation. Therefore, it is impossible to completely rule out the possibility that some patients may have had latent (asymptomatic) distal deep vein thrombosis before the operation. This may lead to the measured preoperative baseline levels of fibrinogen and D-dimer not only reflecting the patient's baseline coagulation status, but also partially overlapping with the fibrinolytic products of existing small thrombi, thereby causing certain interference to the baseline risk assignment of the prediction model. In the future, a prospective design should be utilized to conduct standardized screening for high-risk populations before the operation, in order to further clarify the pure effect relationship between the predictive factors and the new-onset VTE after the operation.

In conclusion, this study successfully developed a prediction tool for VTE occurrence in patients who underwent postoperative orthopedic surgery using a machine learning model. The LGBM, based on preoperative fibrinogen and D-dimer levels, has certain predictive value for VTE risk in the orthopedic surgery population. However, due to the lack of specific variables such as surgical type, duration of surgery, and intraoperative blood loss, its clinical application value in the orthopedic surgery population still needs to be further improved. In the future, the clinical application value of these models needs to be further verified in multicenter, large-sample, and prospective studies.

## Data Availability

The original contributions presented in the study are included in the article/Supplementary Material, further inquiries can be directed to the corresponding author.

## References

[1] Cionac Florescu S Anastase DM Munteanu AM Stoica IC Antonescu D. Venous thromboembolism following major orthopedic surgery. Maedica (Bucur). (2013) 8(2):189–94. 24371484 PMC3865129

[2] Ghulam M Ashfaq Z Ali S Nawaz A Anthony N Ghani U. Association between venous thromboembolism prophylaxis and the incidence of thromboembolism following orthopedic procedures: a cross-sectional study. Cureus. (2024) 16(1):e52652. 10.7759/cureus.52652 38380201 PMC10877209

[3] Izumi M Ikeuchi M Aso K Sugimura N Kamimoto Y Mitani T. Less deep vein thrombosis due to transcutaneous fibular nerve stimulation in total knee arthroplasty: a randomized controlled trial. Knee Surg Sports Traumatol Arthrosc. (2015) 23(11):3317–23. 10.1007/s00167-014-3141-z 24957913

[4] Peng J Wang H Zhang L Lin Z. Construction and efficiency analysis of prediction model for venous thromboembolism risk in the elderly after hip fracture. Zhong Nan Da Xue Xue Bao Yi Xue Ban. (2021) 46(2):142–8. (English, Chinese). 10.11817/j.issn.1672-7347.2021.190722 33678650 PMC10929787

[5] Hu J Zhou Y. The association between lactate dehydrogenase to serum albumin ratio and in-hospital mortality in patients with pulmonary embolism: a retrospective analysis of the MIMIC-IV database. Front Cardiovasc Med. (2024) 11:1398614. 10.3389/fcvm.2024.1398614 38962086 PMC11220285

[6] Johnson AEW Bulgarelli L Shen L Gayles A Shammout A Horng S. Author correction: MIMIC-IV, a freely accessible electronic health record dataset. Sci Data. (2023) 10(1):31. 10.1038/s41597-023-01945-2 36646711 PMC9842744

[7] Guo Y Li L Zheng K Du J Nie J Wang Z. Development and validation of a survival prediction model for patients with advanced non-small cell lung cancer based on LASSO regression. Front Immunol. (2024) 15:1431150. 10.3389/fimmu.2024.1431150 39156899 PMC11327039

[8] Kabir S Pippi Salle JL Chowdhury MEH Abbas TO. Quantification of vesicoureteral reflux using machine learning. J Pediatr Urol. (2024) 20(2):257–64. 10.1016/j.jpurol.2023.10.030 37980211

[9] Jin S Qin D Wang C Liang B Zhang L Gao W. Development, validation, and clinical utility of risk prediction models for cancer-associated venous thromboembolism: a retrospective and prospective cohort study. Asia Pac J Oncol Nurs. (2025) 12:100691. 10.1016/j.apjon.2025.100691 40291141 PMC12032184

[10] Chen HJ Sun LB Kong XY. Research progress of prevention of venous thromboembolism in patients with foot and ankle surgery. Chin J Bone Joint Surg. (2020) 13(11):964–8.

[11] Moisander AM Pamilo K Huopio J Kautiainen H Kuitunen A Paloneva J. The incidence of venous thromboembolism is low when risk stratification-based thromboprophylaxis is used after fast-track hip and knee arthroplasty. BMC Musculoskelet Disord. (2024) 25(1):1094. 10.1186/s12891-024-08256-6 39736658 PMC11687159

[12] Wollenman CC Cox CL Schoenecker JG Wright RW. Venous thromboembolism after knee arthroscopy: incidence, risk factors, prophylaxis, and management. J Am Acad Orthop Surg. (2024) 32(17):e850–60. 10.5435/JAAOS-D-24-00232 39018574

[13] Mula V Parikh S Suresh S Bottle A Loeffler M Alam M. Venous thromboembolism rates after hip and knee arthroplasty and hip fractures. BMC Musculoskelet Disord. (2020) 21(1):95. 10.1186/s12891-020-3100-4 32050949 PMC7017506

[14] Nafee T Gibson CM Travis R Yee MK Kerneis M Chi G. Machine learning to predict venous thrombosis in acutely ill medical patients. Res Pract Thromb Haemost. (2020) 4(2):230–7. 10.1002/rth2.12292 32110753 PMC7040551

[15] Willan J Katz H Keeling D. The use of artificial neural network analysis can improve the risk-stratification of patients presenting with suspected deep vein thrombosis. Br J Haematol. (2019) 185(2):289–96. 10.1111/bjh.15780 30727024

[16] Stevens H Peter K Tran H McFadyen J. Predicting the risk of recurrent venous thromboembolism: current challenges and future opportunities. J Clin Med. (2020) 9(5):1582. 10.3390/jcm9051582 32456008 PMC7290951

[17] Jin J Xu Q Lu J Zhao JY Zhang Q Kong Y. Development of risk prediction model for venous thromboembolism in elderly critically ill patients based on machine learning. Chin Nurs Res. (2025) 39(14):2327–35.

[18] Levin S Toerper M Hamrock E Hinson JS Barnes S Gardner H. Machine-learning-based electronic triage more accurately differentiates patients with respect to clinical outcomes compared with the emergency severity index. Ann Emerg Med. (2018) 71(5):565–74.e2. 10.1016/j.annemergmed.2017.08.005 28888332

[19] Feng XY Tian LY Luo H Li WJ Tan X Yang HF. Application of information technology in nursing care of venous thromboembolism:a literature review. J Nurs Sci. (2022) 37(16):106–10. 10.3870/j.issn.1001-4152.2022.16.106

[20] Wolberg AS Sang Y. Fibrinogen and factor XIII in venous thrombosis and thrombus stability. Arterioscler Thromb Vasc Biol. (2022) 42(8):931–41. 10.1161/ATVBAHA.122.317164 35652333 PMC9339521

[21] Di YJ He J Liu X Wang LS An CY Li XJ. Prediction value of wells score combined with serum fibrinogen and D-dimer in deep venous thrombosis after hip arthroplasty under general anesthesia. Health Med Res Pract. (2025) 22(10):88–93.

[22] Jiao Y Mu X. Coagulation parameters correlate to venous thromboembolism occurrence during the perioperative period in patients with spinal fractures. J Orthop Surg Res. (2023) 18(1):928. 10.1186/s13018-023-04407-y 38057818 PMC10698905

[23] Diaz-Arocutipa C Gonzales-Luna AC Brañez-Condorena A Hernandez AV. Diagnostic accuracy of D-dimer to detect left atrial thrombus in patients with atrial fibrillation: a systematic review and meta-analysis. Heart Rhythm. (2021) 18(12):2128–36. 10.1016/j.hrthm.2021.08.027 34481076

[24] Dong QL Li SN Xiong WR Zhao X. Development of a nomogram model based on multiple factors for predicting risk of venous thromboembolism in patients with traumatic lower extremity fractures. J Guizhou Med Univ. (2026) 51(2):288–95, 304. 10.19367/j.cnki.2096-8388.2026.02.016

[25] Qiao L Yao Y You X Wu D Tsai H Zhou G. Identifying high-risk groups for deep vein thrombosis after primary total knee arthroplasty using preoperative Caprini scores and D-dimer levels. J Orthop Surg Res. (2024) 19(1):616. 10.1186/s13018-024-05074-3 39350206 PMC11443692

[26] Baki H Özçelik İB. Machine learning-based prediction of postoperative deep vein thrombosis following tibial fracture surgery. Diagnostics (Basel). (2025) 15(14):1787. 10.3390/diagnostics15141787 40722536 PMC12293441

[27] Zhang B Qin Y Jiu L Qin C Wang J Zhao H. A study on the risk prediction model for venous thromboembolism in orthopedic inpatients based on machine learning. Front Med (Lausanne). (2025) 12:1574546. 10.3389/fmed.2025.1574546 40641970 PMC12240939

